# Vitamin D Status and Routine Laboratory Data-Derived 25(OH)D Distributional Benchmarks in Adults from Şanlıurfa, Türkiye: Age, Sex, and Seasonal Variation

**DOI:** 10.3390/diagnostics16131995

**Published:** 2026-06-26

**Authors:** Mehmet Akif Bozdayi, Gökhan Çakırca, İsmet Gamze Bozdayi

**Affiliations:** 1 Department of Medical Biochemistry, Mehmet Akif Inan Education and Research Hospital, 63300 Sanliurfa, Türkiye; 2 Department of Medical Biochemistry, Sanliurfa Education and Research Hospital, 63200 Sanliurfa, Türkiye

**Keywords:** 25-hydroxyvitamin D, vitamin D deficiency, distributional interval, routine laboratory data, seasonal variation, Şanlıurfa, Türkiye, laboratory medicine

## Abstract

**Background:** Serum 25-hydroxyvitamin D [25(OH)D] interpretation requires clear distinction between epidemiological thresholds, routine-data distributions, and clinical decision limits. This study evaluated vitamin D status and clinically pre-filtered routine laboratory data-derived 25(OH)D distributional benchmarks among adults from Şanlıurfa, southeastern Türkiye, according to sex, age, and season. **Methods:** This retrospective single-center routine laboratory database study included adults aged 18–65 years tested between 1 January and 31 December 2025, at the Medical Biochemistry Laboratory of Şanlıurfa Mehmet Akif İnan Training and Research Hospital. After eligibility screening, duplicate removal, analytical screening, and predefined clinical pre-filtering, 48,826 participant-level records were analyzed. Serum 25(OH)D was measured using the Elecsys Vitamin D total III electrochemiluminescence immunoassay on a cobas e 801 analyzer/module. The primary distributional estimate was the nonparametric 2.5th–97.5th percentile range. **Results:** Median age was 38 [28–49] years, and 35,043 records were from female participants (71.8%). Median serum 25(OH)D was 12.74 [8.28–19.00] ng/mL. Vitamin D deficiency, severe deficiency, insufficiency, and sufficiency were observed in 38,072 (78.0%), 17,163 (35.2%), 8235 (16.9%), and 2519 (5.2%) records, respectively. Lower 25(OH)D concentrations and higher deficiency prevalence were observed among females, younger adults, and winter/spring samples, with small-to-modest effect magnitudes. The clinically pre-filtered routine-data 2.5th–97.5th percentile range was 3.46–35.50 ng/mL. **Conclusions:** Low 25(OH)D status was widespread among routinely tested adults in Şanlıurfa. The derived range should be interpreted only as a local routine-data distributional benchmark for the tested population, not as a healthy-volunteer reference interval, clinical sufficiency threshold, treatment threshold, or clinical decision limit.

## 1. Introduction

Vitamin D is essential for calcium–phosphate homeostasis, skeletal mineralization, and musculoskeletal health, and inadequate vitamin D status remains a common public health concern across many regions and population groups [1,2,3,4]. Serum 25-hydroxyvitamin D [25(OH)D] is generally regarded as the principal circulating biomarker for assessing vitamin D status because it reflects vitamin D input from cutaneous synthesis, diet, and supplementation more reliably than short-lived active metabolites [5]. However, the interpretation of serum 25(OH)D concentrations is not straightforward, because epidemiological thresholds, clinical decision limits, population-level sufficiency targets, and laboratory-derived reference or distributional intervals are conceptually different constructs [6,7,8].

Commonly used clinical and epidemiological frameworks define vitamin D deficiency as serum 25(OH)D < 20 ng/mL and insufficiency as 20–29.9 ng/mL [6], whereas the Institute of Medicine framework emphasizes that concentrations around 20 ng/mL may meet the needs of most individuals at the population level [7]. More recent guideline perspectives further indicate that outcome-specific 25(OH)D targets for disease prevention remain uncertain and should not be conflated with routine screening thresholds, laboratory reference intervals, or treatment decision limits [8].

Serum 25(OH)D concentrations vary according to demographic, environmental, behavioral, nutritional, and analytical factors. Age, sex, season, latitude, ultraviolet B exposure, skin pigmentation, clothing practices, time spent outdoors, indoor lifestyle, occupational behavior, dietary vitamin D intake, adiposity, supplementation practices, and assay-related variability may all contribute to between-population and within-population differences in measured vitamin D status [3,4,9,10,11]. Therefore, evaluations based only on universal cut-off values may fail to capture locally relevant distributional patterns, particularly in regions where ambient sunlight availability and effective individual ultraviolet B exposure diverge.

Şanlıurfa, located in southeastern Türkiye, has high annual sunlight potential and therefore represents an epidemiologically relevant setting for evaluating serum 25(OH)D distribution [12]. Nevertheless, high ambient sunlight availability does not necessarily ensure adequate vitamin D status at the individual or tested-population level. Effective cutaneous vitamin D synthesis may be reduced by indoor lifestyle, limited skin exposure, occupational patterns, clothing practices, seasonal behavior, dietary intake, adiposity, and supplementation-related differences [4,11,13,14,15]. This apparent “sunlight paradox” is clinically and epidemiologically important: vitamin D deficiency may remain frequent even in environments with favorable sunlight conditions.

Reference intervals are important tools for laboratory result interpretation, but they differ from clinical decision limits and disease-related thresholds. Reference intervals describe the distribution of an analyte in a defined reference population or dataset, whereas clinical decision limits are intended to identify concentrations associated with diagnostic classification, increased risk, clinical intervention, or treatment decisions [16,17,18]. In retrospective laboratory database studies, indirect approaches may estimate intervals from stored routine test results after predefined analytical and clinical filtering [17,18,19]. However, clinically pre-filtered routine laboratory data-derived intervals should not be interpreted as equivalent to prospectively established healthy-volunteer reference intervals or therapeutic decision thresholds [16,17,18,19]. This distinction is especially important for serum 25(OH)D, because a distribution-derived lower limit may reflect a high burden of low vitamin D status in the tested population rather than an optimal biological range.

Indirect and data-driven approaches have become increasingly relevant in laboratory medicine because large routine laboratory databases can provide high statistical precision and real-world insight when direct recruitment of healthy reference individuals is impractical [17,18,19]. Nevertheless, these approaches require the transparent handling of repeated measurements, invalid or analytically implausible results, clinically inappropriate records, missing data, pre-filtering assumptions, and limitations related to testing indication and disease-mixture effects [17,18,19]. Previous Turkish, Mediterranean, and regional studies have reported geographic variability in serum 25(OH)D concentrations and indirect interval estimates [9,20,21,22]. However, evidence remains limited for large-scale, age-, sex-, and season-stratified serum 25(OH)D distributions and clinically pre-filtered routine laboratory data-derived distributional interval estimation in Şanlıurfa.

Therefore, this study aimed to evaluate the distribution of serum 25(OH)D concentrations and the prevalence of vitamin D deficiency, insufficiency, and sufficiency according to age, sex, and season among adults aged 18–65 years undergoing routine laboratory testing in Şanlıurfa, Türkiye. The study further aimed to derive clinically pre-filtered routine laboratory data-derived 25(OH)D distributional intervals using a nonparametric percentile-based approach.

## 2. Materials and Methods

### 2.1. Study Design and Setting

This retrospective, single-center routine laboratory database study evaluated serum 25-hydroxyvitamin D [25(OH)D] concentrations among adults aged 18–65 years who underwent routine laboratory testing in Şanlıurfa, southeastern Türkiye. Serum 25(OH)D records were obtained from the laboratory information system of the Medical Biochemistry Laboratory of Şanlıurfa Mehmet Akif İnan Training and Research Hospital between 1 January and 31 December 2025.

The primary objectives were to describe the distribution of serum 25(OH)D concentrations, estimate the prevalence of vitamin D deficiency, insufficiency, sufficiency, and severe deficiency, evaluate age-, sex-, and season-related variation, and derive clinically pre-filtered routine laboratory data-derived 25(OH)D distributional intervals. The study was not designed as a prospective healthy-volunteer reference interval study, a population-based random-sampling survey, or an interventional study. Accordingly, the derived intervals were interpreted as local routine-data distributional benchmarks, not as healthy-volunteer reference intervals, biological normal ranges, clinical sufficiency thresholds, clinical decision limits, or treatment thresholds.

The reporting of this observational laboratory database study was structured in accordance with the Strengthening the Reporting of Observational Studies in Epidemiology recommendations [23].

### 2.2. Participants and Eligibility Criteria

The data source was the hospital laboratory information system, which contained routinely generated serum 25(OH)D results and the core administrative and analytical variables available for retrospective extraction. The source population consisted of adults aged 18–65 years who had serum 25(OH)D measured as part of routine clinical laboratory evaluation during the study period. Because test ordering was based on routine clinical care, the cohort represents a routinely tested laboratory population and should not be interpreted as a healthy community sample, a randomly selected adult population, or an optimized vitamin D testing cohort.

Eligible records required a valid numeric serum 25(OH)D result reported in ng/mL and complete core analytical variables, including anonymized participant identifier, age, sex, sampling month, season, serum 25(OH)D concentration, age group, and vitamin D status category. When more than one eligible serum 25(OH)D measurement was available for the same participant, only the first eligible record was retained for the participant-level analytical dataset.

**Figure 1:**
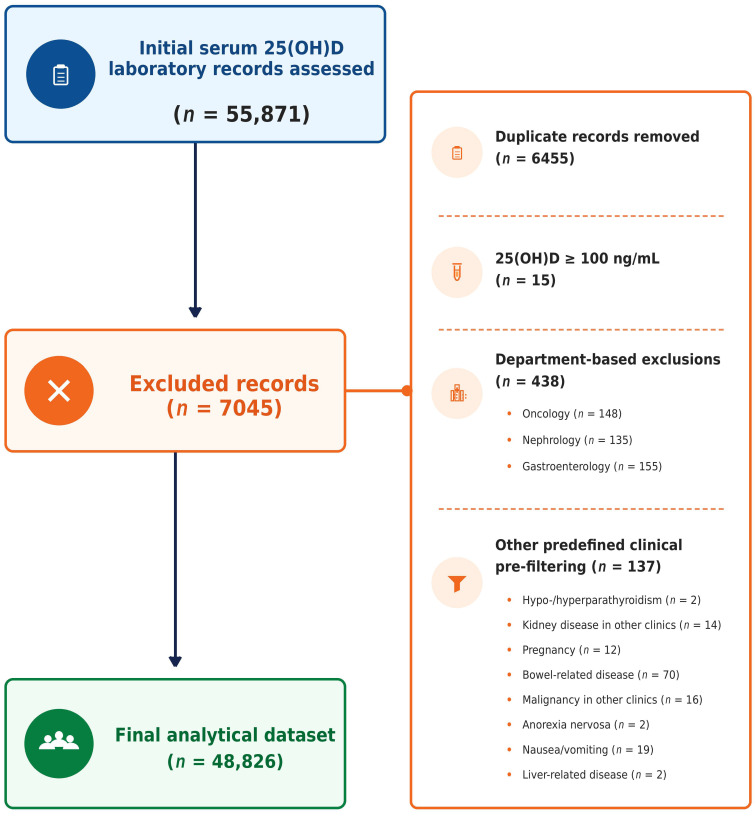
Patient selection and analytical dataset construction flow diagram. 25(OH)D, 25-hydroxyvitamin D; LIS, laboratory information system. Department-based exclusions were applied using requesting-unit information as a conservative clinical pre-filter rather than patient-level clinical adjudication.

As summarized in Figure 1, the flowchart-aligned record-selection process started with 55,871 initial serum 25(OH)D laboratory records. A total of 7045 records were excluded during duplicate removal, analytical screening, and predefined clinical pre-filtering. These exclusions comprised duplicate or repeated records removed according to the first eligible record rule (*n* = 6455), serum 25(OH)D values ≥100 ng/mL excluded as a high-value clinical/epidemiological pre-filter (*n* = 15), department-based exclusions identified using requesting-unit information in the laboratory information system (*n* = 438), and other predefined clinical pre-filtering exclusions (*n* = 137). After the exclusion of 7045 records, the final analytical dataset comprised 48,826 eligible participant-level serum 25(OH)D records.

Variables related to vitamin D supplementation, dietary intake, body mass index, clothing practices, outdoor activity, duration of sunlight exposure, socioeconomic status, urban/rural residence, detailed clinical indication for testing, and detailed comorbidity burden were not available in the laboratory information system and were therefore not included in the primary analyses or stratified reporting.

### 2.3. Eligibility Criteria and Clinical Pre-Filtering

Records were eligible for inclusion if they belonged to adults aged 18–65 years, contained a valid numeric serum 25(OH)D result reported in ng/mL, and had the predefined core analytical variables required for the planned descriptive, comparative, adjusted, and distributional interval analyses. These core variables comprised anonymized participant identifier, age, sex, sampling month, season, serum 25(OH)D concentration, age group, and vitamin D status category.

Records were excluded if they represented repeated measurements beyond the first eligible record for the same participant; contained missing predefined core analytical variables, non-numeric values, unit-inconsistent entries, technically invalid results, or analytically implausible serum 25(OH)D values; had serum 25(OH)D concentrations ≥100 ng/mL; originated from predefined requesting units with a high likelihood of clinically altered vitamin D metabolism; or were associated with predefined clinical contexts likely to materially affect vitamin D metabolism.

In the flowchart-aligned record-selection process, all 7045 excluded records were accounted for by four reported exclusion categories: duplicate or repeated records beyond the first eligible measurement (*n* = 6455), serum 25(OH)D concentrations ≥ 100 ng/mL (*n* = 15), department-based exclusions (*n* = 438), and other predefined clinical pre-filtering exclusions (*n* = 137). Missing predefined core analytical variables, non-numeric results, unit-inconsistent entries, technically invalid results, or analytically implausible serum 25(OH)D values did not constitute an additional stand-alone exclusion category beyond the exclusion categories shown in Figure 1. After completion of eligibility screening and pre-filtering, the final analytical dataset contained no missing values for the predefined core analytical variables.

Department-based exclusions were operationalized using the requesting-unit information recorded in the laboratory information system. These exclusions included records from oncology (*n* = 148), nephrology (*n* = 135), and gastroenterology (*n* = 155) requesting units. This approach was used as a pragmatic conservative clinical pre-filter to reduce the enrichment of the analytical dataset with clinical contexts strongly associated with malignancy, renal disease, malabsorption, gastrointestinal disease, or altered vitamin D metabolism. It should not be interpreted as patient-level diagnostic adjudication or as evidence that every patient from these departments had a vitamin D–altering condition.

The ≥100 ng/mL threshold was applied as a predefined clinical/epidemiological high-value pre-filter to reduce the influence of high-dose supplementation, intoxication-related testing contexts, or atypical high-end clinical observations. This exclusion was not applied on analytical measurement range grounds, because the documented analytical measurement range of the assay extended to 120 ng/mL. Other predefined clinical pre-filtering exclusions included records associated with documented hypo- or hyperparathyroidism (*n* = 2), kidney disease in other clinics (*n* = 14), pregnancy (*n* = 12), bowel-related disease (*n* = 70), malignancy in other clinics (*n* = 16), anorexia nervosa (*n* = 2), nausea/vomiting (*n* = 19), and liver-related disease (*n* = 2), where such information was identifiable in the available source fields.

Because detailed patient-level diagnoses, vitamin D supplementation, dietary intake, body mass index, clothing practices, outdoor activity, duration of sunlight exposure, socioeconomic status, urban/rural residence, detailed comorbidity burden, and clinical indications for testing were not fully available in the laboratory information system, residual clinical heterogeneity and misclassification may have remained in the final analytical dataset despite the predefined pre-filtering strategy.

### 2.4. Measurement of Serum 25(OH)D

Serum 25(OH)D concentrations were measured in serum samples and reported in ng/mL using the Elecsys Vitamin D total III electrochemiluminescence immunoassay on a cobas e 801 analyzer/module (Roche Diagnostics GmbH, Mannheim, Germany; instrument manufactured by Hitachi Instrument [Suzhou], Ltd., Suzhou, China; Elecsys Vitamin D total III reagent catalog number 09038086190). The assay is intended for the in vitro quantitative determination of total 25-hydroxyvitamin D in human serum and plasma and is based on a competitive electrochemiluminescence binding principle on cobas e immunoassay analyzers. The assay was performed, calibrated, and quality controlled according to the manufacturer’s instructions and the laboratory’s routine quality-management procedures [24].

According to the manufacturer’s documentation, the assay is standardized against ID-LC-MS/MS using National Institute of Standards and Technology Standard Reference Material 2972, and the documented analytical measurement range is 3.00–120 ng/mL. The manufacturer-reported lower analytical characteristics include a limit of blank of 2.0 ng/mL, and a limit of detection of 3.0 ng/mL [24,25].

Internal quality control was performed daily before routine sample analysis, and analytical runs were accepted only when quality-control measurements were within the predefined laboratory control limits. External quality assessment was performed monthly through the Bio-Rad External Quality Assurance Services program. Assay documentation, calibration records, analytical measurement range information, manufacturer-provided precision-performance data, and internal/external quality-control records were reviewed before data analysis.

The manufacturer’s package insert reports repeatability coefficients of variation ranging from 1.3% to 6.3% and intermediate precision coefficients of variation ranging from 1.5% to 8.9% for cobas e 801 analyzer (manufactured by Hitachi Instrument [Suzhou], Ltd., Suzhou, China) across evaluated concentration levels [24,25]. These manufacturer-reported performance data were used to document the analytical context of the assay. Lot-specific reagent numbers, calibrator lot details, and lot-specific local quality-control coefficients of variation were not captured in the extracted analytical dataset and were therefore not analyzed as study variables.

No within-study method-comparison experiment against liquid chromatography–tandem mass spectrometry or another reference measurement procedure was performed using the study samples. Therefore, although the assay documentation indicates standardization against ID-LC-MS/MS, the present results should be interpreted within the analytical context of the immunoassay platform used, and direct comparison with LC-MS/MS-based studies or other immunoassay platforms should be made cautiously.

### 2.5. Data Cleaning, Duplicate Handling, and Missing Data

All extracted records were evaluated for data integrity, duplicate measurements, analytical validity, unit consistency, missing predefined core variables, and predefined clinical exclusion criteria before the construction of the final participant-level analytical dataset. Duplicate control was performed using the anonymized participant identifier, and only the first eligible serum 25(OH)D measurement for each participant was retained. Records beyond the first eligible measurement were excluded as duplicate or repeated records.

The flowchart-aligned record-selection process accounted for all 7045 excluded records in four reported exclusion categories: duplicate or repeated records beyond the first eligible measurement (*n* = 6455), serum 25(OH)D concentrations ≥100 ng/mL (*n* = 15), department-based exclusions (*n* = 438), and other predefined clinical pre-filtering exclusions (*n* = 137). Missing predefined core variables, non-numeric values, unit-inconsistent entries, technically invalid results, and analytically implausible serum 25(OH)D values were assessed during eligibility screening and did not constitute an additional stand-alone exclusion category beyond the exclusion categories shown in Figure 1.

No missing values were present in the final analytical dataset for the predefined core analytical variables, including anonymized participant identifier, age, sex, sampling month, season, serum 25(OH)D concentration, age group, and vitamin D status category. This statement applies only to the final cleaned analytical dataset after eligibility screening, duplicate handling, analytical screening, and predefined clinical pre-filtering, and should not be interpreted as indicating that the unfiltered source laboratory database contained no missing data. Seasonal classification was based on the available sampling-month variable.

After predefined screening, serum 25(OH)D concentrations in the final analytical dataset ranged from 3.00 to 99.90 ng/mL. The final analytical dataset was used for all primary descriptive, comparative, adjusted, and distributional interval analyses.

### 2.6. Age, Sex, and Seasonal Grouping

Sex was categorized as female or male according to laboratory information system records. Age was evaluated both as a continuous variable and as a categorical variable using the predefined age groups of 18–35 years, 36–50 years, and 51–65 years. These age groups were selected to provide clinically interpretable adult life-stage strata while preserving adequate subgroup size for sex-, age-, and season-stratified descriptive and distributional analyses. Sampling month was used to define seasons as follows: winter, December–February; spring, March–May; summer, June–August; and autumn, September–November.

### 2.7. Vitamin D Status Classification

Vitamin D status was classified using predefined epidemiological thresholds based on serum 25(OH)D concentration. Vitamin D deficiency was defined as <20 ng/mL, insufficiency as 20–29.9 ng/mL, and sufficiency as ≥30 ng/mL, consistent with commonly used clinical and epidemiological cut-offs [6]. Severe vitamin D deficiency was defined as serum 25(OH)D < 10 ng/mL.

These thresholds were used only for epidemiological classification and prevalence estimation. They were not treated as laboratory-derived distributional limits, healthy-volunteer reference limits, clinical decision limits, or treatment thresholds. Conversely, the routine laboratory data-derived distributional intervals estimated in this study were not interpreted as vitamin D sufficiency thresholds or therapeutic targets.

### 2.8. Routine Laboratory Data-Derived Distributional Interval Estimation

The primary interval estimate was defined as the nonparametric 2.5th–97.5th percentile distributional interval. Lower and upper distributional limits were estimated using the nonparametric percentile method, and 95% confidence intervals for the lower and upper limits were calculated using distribution-free order-statistic procedures. The 95% confidence level was retained to maintain consistency with the reporting of prevalence estimates, adjusted associations, and interval estimates throughout the manuscript; however, these intervals were interpreted as routine-data distributional benchmarks rather than clinical decision thresholds.

To assess the robustness of the primary nonparametric distributional limits, a Tukey 1.5 × IQR sensitivity analysis was performed for the overall dataset. Tukey fences were calculated from the overall serum 25(OH)D distribution as Q1 − 1.5 × IQR and Q3 + 1.5 × IQR. Observations outside these fences were excluded only for the sensitivity interval calculation. The Tukey-cleaned dataset was not used as the primary analytical dataset and did not replace the full clinically pre-filtered dataset for descriptive, comparative, adjusted, or subgroup-specific analyses. This approach was used only as a distributional robustness check for the nonparametric 2.5th–97.5th percentile limits.

Although percentile-based laboratory medicine methodology was used to calculate the distributional limits, this study was not a prospective CLSI-style healthy-volunteer reference interval study. Because this was a retrospective routine laboratory data-derived analysis rather than a prospective healthy-volunteer reference interval study, the derived limits were interpreted as local routine-data distributional benchmarks and not as clinical sufficiency thresholds, treatment decision limits, or evidence of biological optimality.

Distributional intervals were calculated for the overall analytical dataset and for prespecified subgroups according to sex, age group, and season.

### 2.9. Statistical Analysis

All primary descriptive, comparative, and regression analyses were performed using IBM SPSS Statistics for Windows, version 27.0 (IBM Corp., Armonk, NY, USA). Supplementary calculations not directly implemented in standard SPSS menu-based procedures, including distribution-free order-statistic confidence intervals for nonparametric distributional limits and Tukey 1.5 × IQR sensitivity calculations, were performed using Microsoft Excel for Microsoft 365 from the exported cleaned analytical dataset. These supplementary calculations were used only for interval estimation and sensitivity assessment and did not replace the predefined final analytical dataset. All statistical procedures were interpreted in accordance with the retrospective routine laboratory database design.

The distribution of continuous variables was assessed using the Shapiro–Wilk test, histograms, Q–Q plots, and boxplots. Normally distributed continuous variables were summarized as mean ± standard deviation, whereas non-normally distributed variables were summarized as median [IQR]. Categorical variables were summarized as n (%).

Because serum 25(OH)D concentrations were non-normally distributed, sex-based comparisons were performed using the Mann–Whitney U test, whereas comparisons across age groups and seasons were performed using the Kruskal–Wallis test. Pairwise post hoc comparisons were adjusted using Bonferroni or Dunn–Bonferroni procedures, as appropriate. Vitamin D status categories were compared using Pearson’s χ^2^ test; Fisher’s exact test or the Fisher–Freeman–Halton exact test was planned for contingency tables with insufficient expected cell counts.

Prevalence estimates were reported as n (%) with Wilson 95% confidence intervals. Effect sizes were reported to avoid overinterpreting statistical significance in this large dataset: r for Mann–Whitney U tests, epsilon-squared for Kruskal–Wallis tests, and Cramér’s V for χ^2^ tests. Adjusted associations with serum 25(OH)D concentration were evaluated using natural-log-transformed serum 25(OH)D models and reported as geometric mean ratios with 95% confidence intervals. Vitamin D deficiency was evaluated using binary logistic regression and reported as adjusted odds ratios with 95% confidence intervals. The adjusted models included sex, age group, and season as independent variables.

All statistical tests were two-sided, and α = 0.05 was used as the statistical significance threshold. Multiplicity correction was applied for post hoc comparisons. All regression and subgroup analyses were interpreted as adjusted associations within a routine laboratory-tested population and not as causal effects.

### 2.10. Ethical Considerations

This study was approved by the Clinical Research Ethics Committee of Gaziantep Islam Science and Technology University (Decision No: 2026-2ÖNP-0035; Date: 21 April 2026). Because the study was retrospective and used anonymized routine laboratory data, the requirement for informed consent was waived by the ethics committee. All analytical data were anonymized before analysis, and data handling was conducted in accordance with institutional confidentiality requirements and the Declaration of Helsinki.

## 3. Results

### 3.1. Study Population and Analytical Dataset

As shown in Figure 1, after eligibility screening, data cleaning, duplicate handling, analytical screening, and predefined clinical pre-filtering, the final analytical dataset comprised 48,826 eligible participant-level serum 25(OH)D records. The record-selection process began with 55,871 initial laboratory records, of which 7045 were excluded. Exclusions included duplicate or repeated measurements beyond the first eligible record (*n* = 6455), serum 25(OH)D concentrations ≥ 100 ng/mL (*n* = 15), department-based exclusions (*n* = 438), and other predefined clinical pre-filtering exclusions (*n* = 137).

The median age was 38 [28–49] years, and the mean age was 38.79 ± 13.26 years. Female participants accounted for 35,043 records (71.8%), and male participants accounted for 13,783 records (28.2%). The age-group distribution was 22,084 records (45.2%) for 18–35 years, 15,598 (31.9%) for 36–50 years, and 11,144 (22.8%) for 51–65 years. Seasonal distribution was balanced across winter, spring, summer, and autumn. No missing values were present for the predefined core analytical variables in the final analytical dataset. Descriptive characteristics are summarized in Table 1.

### 3.2. Overall Serum 25(OH)D Distribution and Vitamin D Status

As summarized in Table 1, serum 25(OH)D concentrations showed a right-skewed distribution. The overall median serum 25(OH)D concentration was 12.74 [8.28–19.00] ng/mL, with a mean ± standard deviation of 14.59 ± 8.69 ng/mL and an observed range of 3.00–99.90 ng/mL.

Using the predefined epidemiological thresholds shown in Table 1, vitamin D deficiency, defined as serum 25(OH)D < 20 ng/mL, was observed in 38,072 records (78.0%). Severe vitamin D deficiency, defined as serum 25(OH)D < 10 ng/mL, was observed in 17,163 records (35.2%). Vitamin D insufficiency and sufficiency were observed in 8235 (16.9%) and 2519 (5.2%) records, respectively.

### 3.3. Sex-, Age-, and Season-Specific Differences

As shown in Table 2, serum 25(OH)D concentrations differed by sex, with lower concentrations in females than males: 11.43 [7.48–18.00] ng/mL versus 15.61 [11.30–20.70] ng/mL, respectively. The sex-based comparison was statistically significant (Mann–Whitney U = 175,320,412.0; *p* < 0.001), with a small-to-moderate effect size for serum 25(OH)D concentration (r = −0.214) and a small effect size for vitamin D status distribution (Cramér’s V = 0.106). Vitamin D deficiency was more frequent in females than males (80.3% vs. 72.1%).

As shown in Table 2, serum 25(OH)D concentrations also differed across predefined age groups (Kruskal–Wallis H = 635.18; *p* < 0.001). Median serum 25(OH)D concentrations increased across age strata, from 11.80 [7.75–17.71] ng/mL in the 18–35-year group to 13.20 [8.58–19.40] ng/mL in the 36–50-year group and 14.16 [9.20–20.90] ng/mL in the 51–65-year group. Post hoc grouping showed that all three age groups differed from one another after multiplicity correction. Vitamin D deficiency prevalence decreased across the same age strata, from 81.8% to 76.7% and 72.2%, respectively. Although age-group differences were statistically significant, the effect sizes were small for both serum 25(OH)D concentration (ε^2^ = 0.013) and vitamin D status distribution (Cramér’s V = 0.071).

As shown in Table 2, seasonal variation was observed for serum 25(OH)D concentrations (Kruskal–Wallis H = 1000.66; *p* < 0.001). Median serum 25(OH)D concentrations were lowest in spring and winter, 11.40 [7.38–17.60] ng/mL and 11.64 [7.77–17.40] ng/mL, respectively, and higher in autumn and summer, 13.70 [8.84–19.80] ng/mL and 14.50 [9.53–20.50] ng/mL, respectively. Post hoc grouping indicated that winter and spring were statistically similar, autumn formed an intermediate group, and summer had the highest serum 25(OH)D concentrations. Vitamin D deficiency prevalence was highest in spring (81.7%) and winter (81.4%), followed by autumn (75.7%) and summer (73.1%). Season-related effect sizes were small for both serum 25(OH)D concentration (ε^2^ = 0.020) and vitamin D status distribution (Cramér’s V = 0.067), indicating that statistical significance should be interpreted in the context of the large sample size.

**Figure 2:**
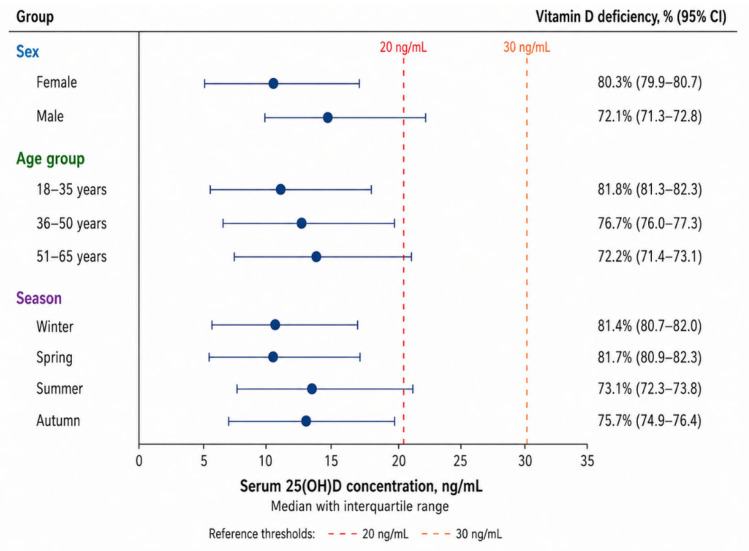
Main subgroup patterns in serum 25(OH)D concentration and vitamin D deficiency prevalence. Median serum 25(OH)D concentrations are shown with interquartile ranges according to sex, age group, and season. Vitamin D deficiency prevalence, defined as serum 25(OH)D < 20 ng/mL, is shown with Wilson 95% confidence intervals. Dashed vertical reference lines indicate the commonly used epidemiological thresholds of 20 ng/mL and 30 ng/mL and should not be interpreted as distributional interval limits. 25(OH)D, 25-hydroxyvitamin D; CI, confidence interval; IQR, interquartile range.

Figure 2 summarizes the main subgroup patterns in serum 25(OH)D concentration and vitamin D deficiency prevalence. Overall, females, younger adults, and winter/spring samples showed lower median serum 25(OH)D concentrations and higher vitamin D deficiency prevalence. However, the corresponding effect sizes were small or small-to-modest; therefore, these subgroup differences should be interpreted primarily in terms of direction, magnitude, confidence intervals, and epidemiological context rather than *p*-values alone.

### 3.4. Routine Laboratory Data-Derived 25(OH)D Distributional Intervals

As presented in Table 3, the overall routine laboratory data-derived 25(OH)D distributional interval, estimated using the nonparametric 2.5th–97.5th percentile approach, was 3.46–35.50 ng/mL. The 95% confidence interval was 3.39–3.51 ng/mL for the lower distributional limit and 35.01–36.00 ng/mL for the upper distributional limit. These values describe the distribution of the clinically pre-filtered routine laboratory dataset and should not be interpreted as healthy-volunteer reference limits, clinical sufficiency thresholds, treatment thresholds, or clinical decision limits.

In the Tukey 1.5 × IQR sensitivity analysis, which was restricted to robustness assessment of the overall distributional interval, the Tukey-cleaned dataset comprised 47,540 records. The corresponding sensitivity distributional interval was 3.43–30.10 ng/mL. The lower distributional limit was nearly unchanged compared with the primary analysis, whereas the upper distributional limit was lower after exclusion of high-end Tukey-flagged observations. This sensitivity analysis did not replace the primary clinically pre-filtered analysis and was not used as the primary analytical dataset.

As shown in Table 3, the lower distributional limit was higher in males than females (5.31 vs. 3.18 ng/mL), while the upper distributional limit was slightly higher in females than males (36.06 vs. 33.97 ng/mL). Across age strata, the lower distributional limits ranged from 3.26 ng/mL in the 18–35-year group to 3.78 ng/mL in the 51–65-year group, while upper limits ranged from 32.80 to 39.63 ng/mL. Across seasons, lower limits ranged from 3.25 ng/mL in winter to 4.11 ng/mL in summer, and upper limits were broadly similar across seasonal strata, ranging from 34.92 to 35.60 ng/mL.

### 3.5. Adjusted Associations with Serum 25(OH)D Concentration and Vitamin D Deficiency

As summarized in Table 4, in the adjusted natural-log-transformed serum 25(OH)D model, female sex was associated with lower serum 25(OH)D concentration compared with male sex (GMR = 0.769; 95% CI, 0.760–0.778; *p* < 0.001). Using the 51–65-year group as the reference, the 18–35-year group (GMR = 0.855; 95% CI, 0.844–0.866; *p* < 0.001) and the 36–50-year group (GMR = 0.940; 95% CI, 0.927–0.953; *p* < 0.001) had lower adjusted serum 25(OH)D concentrations. Compared with summer, winter (GMR = 0.833; 95% CI, 0.821–0.845; *p* < 0.001), spring (GMR = 0.820; 95% CI, 0.808–0.832; *p* < 0.001), and autumn (GMR = 0.933; 95% CI, 0.920–0.947; *p* < 0.001) were associated with lower adjusted serum 25(OH)D concentrations.

As summarized in Table 4, in the binary logistic regression model for vitamin D deficiency, female sex was associated with higher odds of deficiency compared with male sex (adjusted OR = 1.56; 95% CI, 1.49–1.63; *p* < 0.001). Compared with the 51–65-year group, the odds of deficiency were higher in the 18–35-year group (adjusted OR = 1.72; 95% CI, 1.63–1.82; *p* < 0.001) and the 36–50-year group (adjusted OR = 1.25; 95% CI, 1.18–1.32; *p* < 0.001). Compared with summer, the odds of deficiency were higher in winter (adjusted OR = 1.65; 95% CI, 1.56–1.76; *p* < 0.001), spring (adjusted OR = 1.68; 95% CI, 1.58–1.79; *p* < 0.001), and autumn (adjusted OR = 1.16; 95% CI, 1.09–1.23; *p* < 0.001). These models describe adjusted associations within the routine laboratory-tested population and should not be interpreted as causal effects.

## 4. Discussion

### 4.1. Principal Findings

This large retrospective routine laboratory database study of 48,826 adults aged 18–65 years from Şanlıurfa, Türkiye, demonstrated a right-skewed serum 25(OH)D distribution, a low overall median concentration, and a high burden of vitamin D deficiency within a routinely tested adult laboratory population. Using the predefined epidemiological threshold of <20 ng/mL, vitamin D deficiency was observed in 78.0% of records, while severe deficiency, insufficiency, and sufficiency were observed in 35.2%, 16.9%, and 5.2%, respectively. Serum 25(OH)D concentrations differed by sex, age group, and season: lower concentrations and higher deficiency prevalence were observed among females, younger adults, and winter/spring samples. The adjusted models supported these descriptive patterns, although all associations should be interpreted as adjusted associations rather than causal effects.

The overall nonparametric 2.5th–97.5th percentile distributional interval was 3.46–35.50 ng/mL. This interval should be interpreted as a clinically pre-filtered routine laboratory data-derived distributional benchmark, not as a healthy-volunteer reference interval, clinical sufficiency threshold, treatment threshold, or clinical decision limit. The very low lower distributional limit mainly reflects the high burden of low measured 25(OH)D concentrations in the tested population and should not be interpreted as evidence that very low 25(OH)D values are clinically acceptable.

### 4.2. Interpretation of the High Deficiency Burden in a High-Sunlight Region

The high prevalence of low vitamin D status in this study is consistent with the broader literature showing that vitamin D deficiency remains common across diverse populations and varies substantially by geography, demographic structure, lifestyle, season, assay platform, and threshold definition [1,2,3,4,10]. Large-scale global and European analyses have demonstrated marked regional variation in serum 25(OH)D concentrations, supporting locally contextualized interpretation rather than assuming that ambient sunlight availability alone predicts vitamin D adequacy [3,10].

The present findings are also broadly consistent with Turkish and regional studies reporting frequent vitamin D deficiency, although prevalence estimates differ according to study design, age structure, clinical setting, season, assay method, supplementation behavior, and the threshold used to define deficiency [13,26,27,28,29,30,31]. Therefore, the present data should not be interpreted as a direct population-representative estimate for all adults living in Şanlıurfa; rather, they provide a large-scale estimate among adults undergoing routine laboratory testing in a regional healthcare setting.

Şanlıurfa has high annual sunlight potential [12]. However, the present findings reinforce the concept that high-ambient sunlight does not necessarily translate into adequate individual serum 25(OH)D concentrations. Effective cutaneous vitamin D synthesis depends on biologically effective UVB exposure and may be modified by season, latitude, time spent outdoors, clothing practices, occupational patterns, skin pigmentation, dietary intake, adiposity, supplementation, and sociocultural factors [4,11,13,14,15,32,33,34]. Because these behavioral, nutritional, anthropometric, and socioeconomic variables were not available in the present routine laboratory dataset, the observed high deficiency prevalence should be interpreted as an epidemiological signal rather than evidence of a specific causal mechanism.

### 4.3. Sex-, Age-, and Season-Related Variation

Female participants had lower serum 25(OH)D concentrations and higher vitamin D deficiency prevalence than male participants. Similar sex-related patterns have been reported in studies from Türkiye and the Middle East [13,14,15,30,32,33,34]. Potential explanations include differences in clothing practices, direct sun exposure, indoor lifestyle, body composition, dietary intake, supplementation behavior, healthcare-seeking behavior, and sociocultural determinants. However, these mechanisms were not directly measured in the present dataset. Therefore, the observed sex-related difference should be interpreted as an adjusted association within a routinely tested laboratory population rather than evidence of biological or behavioral causality.

An age-related gradient was also observed. Younger adults had lower median serum 25(OH)D concentrations and higher vitamin D deficiency prevalence than participants aged 51–65 years. This pattern is compatible with Turkish routine-data and population-based studies showing that serum 25(OH)D concentrations may vary according to age, sex, season, region, healthcare setting, and testing context [26,27,28,29,30,31]. However, because supplementation use, dietary vitamin D intake, outdoor activity, clothing practices, body mass index, and clinical indication for testing were not available in the laboratory information system, the age-related pattern observed in the present study should not be interpreted as a biological age effect. The higher serum 25(OH)D concentrations observed in older adults may reflect differences in supplementation, clinical monitoring, preventive health behavior, outdoor activity, occupation, diet, or testing patterns, none of which could be directly evaluated in the extracted laboratory dataset.

Seasonal variation was evident, with lower serum 25(OH)D concentrations and higher deficiency prevalence in winter and spring and relatively higher concentrations in summer and autumn. This pattern is biologically plausible because UVB-dependent cutaneous vitamin D synthesis varies according to season, latitude, solar zenith angle, and effective skin exposure [4,11]. The similarity between winter and spring may reflect delayed recovery of serum 25(OH)D after winter, limited outdoor exposure, clothing-related reduction in skin exposure, indoor occupational patterns, or other unmeasured seasonal behaviors. Thus, even in a high-sunlight region such as Şanlıurfa, seasonal and behavioral determinants may limit effective UVB exposure sufficiently to produce measurable variation in serum 25(OH)D concentrations.

### 4.4. Interpretation of Routine Laboratory Data-Derived Distributional Intervals

The overall clinically pre-filtered routine laboratory data-derived distributional interval was 3.46–35.50 ng/mL. This interval was calculated using the nonparametric percentile approach, but it should not be interpreted as a healthy-volunteer reference interval. The dataset was derived from routine laboratory testing, and the analyzed cohort was not prospectively recruited as a healthy reference population. Therefore, the derived interval describes the local distribution of serum 25(OH)D values after analytical and clinical pre-filtering rather than a biological normal range.

The distinction between clinical vitamin D thresholds and laboratory-derived distributional limits is central to the interpretation of this study. Vitamin D deficiency, insufficiency, and sufficiency categories were defined using predefined epidemiological thresholds [6,7]. In contrast, the routine laboratory data-derived interval was based on the observed percentile distribution of serum 25(OH)D in the clinically pre-filtered dataset. These constructs should not be used interchangeably. In a population with a high burden of low 25(OH)D status, a distribution-derived lower limit may primarily reflect the local distribution of measured values rather than an optimal physiological range or a clinically acceptable lower boundary.

The Tukey 1.5 × IQR sensitivity analysis yielded a similar lower distributional limit but a narrower upper distributional limit after exclusion of high-end Tukey-flagged observations. This finding suggests that the lower tail of the distribution was not materially affected by Tukey-based exclusion, whereas high-end routine laboratory observations influenced the upper percentile estimate. However, the Tukey rule is a data-driven outlier-screening procedure rather than a clinical exclusion criterion. Therefore, the Tukey-cleaned dataset was retained only as a sensitivity analysis and was not used as the primary analytical dataset.

### 4.5. Adjusted Association Interpretation

Although sex-, age-, and season-related comparisons were statistically significant, the corresponding effect sizes were generally small or small-to-modest. This distinction is important because very large datasets can produce highly significant *p*-values even when absolute differences or standardized effect magnitudes are modest. The adjusted analyses supported the descriptive patterns, with female sex, younger age groups, and non-summer seasons associated with lower serum 25(OH)D concentrations and higher odds of vitamin D deficiency. However, these findings should be interpreted as adjusted associations rather than causal effects. Residual confounding by unmeasured variables, including supplementation, dietary intake, body mass index, clothing practices, outdoor activity, duration of sunlight exposure, socioeconomic status, comorbidities, and indication for testing, cannot be excluded. Therefore, the practical interpretation of the findings should emphasize direction, magnitude, confidence intervals, clinical plausibility, and the limitations of the routine laboratory-tested design rather than statistical significance alone.

### 4.6. Strengths and Limitations

This study has several strengths. First, the large sample size provided high statistical precision for estimating serum 25(OH)D distributions, deficiency prevalence, and distributional limits in a regional adult routine laboratory population. Second, the study covered a full calendar year, enabling the evaluation of seasonal variation across winter, spring, summer, and autumn. Third, the analysis combined epidemiological classification of vitamin D status with nonparametric distributional interval estimation, subgroup-specific descriptive reporting, effect-size estimation, and adjusted association modeling. Fourth, reporting of effect sizes, 95% confidence intervals, post hoc comparisons, and adjusted associations improved interpretability. Finally, the explicit distinction between clinical vitamin D thresholds and routine laboratory data-derived distributional intervals is an important methodological strength for laboratory medicine interpretation.

Several limitations should also be acknowledged. The retrospective, single-center routine laboratory database design limits causal inference and may restrict generalizability to the wider adult population of Şanlıurfa or to other regions. The cohort consisted of individuals who underwent serum 25(OH)D testing as part of routine clinical care; therefore, selection bias, healthcare-seeking bias, and testing-indication bias are possible. Although duplicate control, analytical filtering, and predefined clinical exclusions were applied, the dataset does not represent a prospectively recruited healthy reference population. Department-based exclusions were applied using requesting-unit information as a conservative clinical pre-filter; therefore, some patients may have been excluded because of departmental affiliation rather than individually verified clinical conditions, and residual clinical heterogeneity may have remained in the analytical dataset. Information on vitamin D supplementation, dietary intake, body mass index, clothing practices, outdoor activity, duration of sunlight exposure, socioeconomic status, urban/rural residence, detailed comorbidity status, and detailed clinical indication for testing was unavailable. This limitation is particularly important when interpreting the higher serum 25(OH)D concentrations observed among older adults, because supplementation behavior, clinical monitoring, preventive health practices, occupational exposure, diet, and healthcare-seeking patterns could not be directly evaluated. Analytical variation related to immunoassay-based measurement should also be considered when comparing these results with studies using other immunoassay platforms or LC-MS/MS-based reference measurement procedures.

### 4.7. Clinical, Public Health, and Laboratory Implications

The findings indicate a high burden of low serum 25(OH)D concentrations among adults undergoing routine laboratory testing in Şanlıurfa, despite the region’s high sunlight potential. From an epidemiological and public health perspective, these results support locally contextualized awareness regarding effective sunlight exposure, dietary vitamin D sources, and guideline-consistent supplementation in clinically indicated groups, rather than the indiscriminate interpretation of high-ambient sunlight as adequate vitamin D status [4,8]. Because supplementation, diet, body mass index, clothing practices, outdoor activity, and clinical indication for testing were unavailable, the present study cannot define individual-level preventive or therapeutic strategies.

From a laboratory medicine perspective, the study illustrates both the value and the limitations of clinically pre-filtered routine laboratory datasets for describing analyte distributions and estimating local distributional benchmarks when direct prospective reference sampling is not feasible [16,17,18,19]. Routine laboratory data-derived distributional intervals should complement, not replace, established clinical thresholds, patient-level risk assessment, assay-specific analytical interpretation, and guideline-based clinical judgment. Future multicenter studies incorporating supplementation, dietary intake, body composition, clothing practices, outdoor activity, standardized analytical methods, urban/rural residence, and clinical indications for testing would help clarify the behavioral, clinical, and methodological factors underlying the observed deficiency burden.

## 5. Conclusions

In this large retrospective routine laboratory database study of adults aged 18–65 years in Şanlıurfa, Türkiye, low serum 25(OH)D status was widespread among routinely tested adults, and vitamin D deficiency was highly prevalent despite the region’s high sunlight potential. Serum 25(OH)D concentrations and vitamin D deficiency prevalence differed according to sex, age group, and season, with lower concentrations and higher deficiency prevalence observed among females, younger adults, and winter/spring samples. However, these differences should be interpreted in light of generally small-to-modest effect magnitudes, the retrospective routine laboratory-tested design, and the absence of individual-level data on supplementation, dietary intake, clothing practices, sunlight exposure, body mass index, and testing indication.

The clinically pre-filtered routine laboratory data-derived nonparametric 25(OH)D distributional range provides a local distributional benchmark for the tested population. It should not be interpreted as a healthy-volunteer reference interval, biological normal range, clinical sufficiency threshold, treatment threshold, or clinical decision limit. These findings support the locally contextualized interpretation of serum 25(OH)D results and highlight both the epidemiological value and the methodological limitations of large routine laboratory datasets for laboratory medicine research. Future multicenter studies incorporating supplementation, dietary intake, body mass index, clothing practices, outdoor activity, sunlight exposure, urban/rural residence, standardized analytical methods, and clinical indications for testing are needed to clarify the behavioral, clinical, and analytical factors underlying the observed distributional patterns.

## Figures and Tables

**Table 1:** Baseline characteristics of the study population and overall serum 25(OH)D distribution.

Variable	Overall Cohort
Primary analytical dataset, n	48,826
Age, years	38 [28–49]
Age, mean ± SD, years	38.79 ± 13.26
Age range, years	18–65
Female, *n* (%)	35,043 (71.8)
Male, *n* (%)	13,783 (28.2)
Age group 18–35 years, *n* (%)	22,084 (45.2)
Age group 36–50 years, *n* (%)	15,598 (31.9)
Age group 51–65 years, *n* (%)	11,144 (22.8)
Winter samples, *n* (%)	13,076 (26.8)
Spring samples, *n* (%)	11,674 (23.9)
Summer samples, *n* (%)	12,192 (25.0)
Autumn samples, *n* (%)	11,884 (24.3)
Serum 25(OH)D, ng/mL	12.74 [8.28–19.00]
Serum 25(OH)D, mean ± SD, ng/mL	14.59 ± 8.69
Serum 25(OH)D range, ng/mL	3.00–99.90
Severe vitamin D deficiency < 10 ng/mL, *n* (%)	17,163 (35.2)
Vitamin D deficiency < 20 ng/mL, *n* (%)	38,072 (78.0)
Vitamin D insufficiency 20–29.9 ng/mL, *n* (%)	8235 (16.9)
Vitamin D sufficiency ≥ 30 ng/mL, *n* (%)	2519 (5.2)
Missing data for predefined core analytical variables in the final analytical dataset, n	0

Data are presented as median [IQR], mean ± standard deviation, or n (%), as appropriate. The statement of zero missing data refers only to the final analytical dataset after exclusion of records with missing predefined core analytical variables. Core analytical variables comprised age, sex, sampling month, season, serum 25(OH)D concentration, age group, and vitamin D status category. Vitamin D deficiency was defined as serum 25(OH)D < 20 ng/mL, insufficiency as 20–29.9 ng/mL, sufficiency as ≥30 ng/mL, and severe deficiency as <10 ng/mL. 25(OH)D, 25-hydroxyvitamin D; IQR, interquartile range; SD, standard deviation.

**Table 2:** Serum 25(OH)D concentrations and vitamin D status by sex, age group, and season.

Variable	Subgroup	*n*	Serum 25(OH)D, Median [IQR]	Mean ± SD	Deficiency n/N (%; 95% CI)	Insufficiency *n* (%)	Sufficiency *n* (%)	Global *p* Value	Effect Size	Post Hoc Group
Sex	Female	35,043	11.43 [7.48–18.00]	13.79 ± 8.89	28,134/35,043 (80.3; 79.9–80.7)	5035 (14.4)	1874 (5.3)	<0.001	r = −0.214; V = 0.106	—
	Male	13,783	15.61 [11.30–20.70]	16.64 ± 7.78	9938/13,783 (72.1; 71.3–72.8)	3200 (23.2)	645 (4.7)			—
Age group	18–35 years	22,084	11.80 [7.75–17.71]	13.62 ± 8.20	18,062/22,084 (81.8; 81.3–82.3)	3213 (14.5)	809 (3.7)	<0.001	ε^2^ = 0.013; V = 0.071	a
	36–50 years	15,598	13.20 [8.58–19.40]	14.91 ± 8.61	11,960/15,598 (76.7; 76.0–77.3)	2806 (18.0)	832 (5.3)			b
	51–65 years	11,144	14.16 [9.20–20.90]	16.08 ± 9.46	8050/11,144 (72.2; 71.4–73.1)	2216 (19.9)	878 (7.9)			c
Season	Winter	13,076	11.64 [7.77–17.40]	13.77 ± 8.70	10,641/13,076 (81.4; 80.7–82.0)	1768 (13.5)	667 (5.1)	<0.001	ε^2^ = 0.020; V = 0.067	a
	Spring	11,674	11.40 [7.38–17.60]	13.59 ± 8.72	9532/11,674 (81.7; 80.9–82.3)	1598 (13.7)	544 (4.7)			a
	Summer	12,192	14.50 [9.53–20.50]	15.87 ± 8.52	8908/12,192 (73.1; 72.3–73.8)	2605 (21.4)	679 (5.6)			c
	Autumn	11,884	13.70 [8.84–19.80]	15.17 ± 8.60	8991/11,884 (75.7; 74.9–76.4)	2264 (19.1)	629 (5.3)			b

Serum 25(OH)D concentrations were compared using the Mann–Whitney U test for sex and the Kruskal–Wallis test for age group and season. Vitamin D status categories were compared using Pearson’s χ^2^ test. Deficiency prevalence is reported with Wilson 95% confidence intervals. Effect sizes are reported as r for the Mann–Whitney U test, epsilon-squared for Kruskal–Wallis tests, and Cramér’s V for categorical comparisons. Given the large sample size, statistical significance should be interpreted together with the magnitude of the corresponding effect sizes. Post hoc letters indicate multiplicity-adjusted pairwise comparisons of serum 25(OH)D concentrations within each variable; subgroups sharing the same letter did not differ significantly, whereas different letters indicate statistically significant differences after correction. 25(OH)D, 25-hydroxyvitamin D; CI, confidence interval; IQR, interquartile range; SD, standard deviation.

**Table 3:** Nonparametric routine laboratory data-derived serum 25(OH)D distributional intervals.

Group	*n*	Median [IQR], ng/mL	Lower Limit, 2.5th Percentile	95% CI for Lower Limit	Upper Limit, 97.5th Percentile	95% CI for Upper Limit	Analysis Status
Overall	48,826	12.74 [8.28–19.00]	3.46	3.39–3.51	35.50	35.01–36.00	Primary distributional interval
Overall after Tukey 1.5 × IQR exclusion	47,540	12.50 [8.18–18.40]	3.43	3.36–3.48	30.10	29.90–30.30	Sensitivity analysis
Female	35,043	11.43 [7.48–18.00]	3.18	3.10–3.25	36.06	35.50–36.56	Subgroup distributional interval
Male	13,783	15.61 [11.30–20.70]	5.31	5.11–5.46	33.97	33.33–34.90	Subgroup distributional interval
18–35 years	22,084	11.80 [7.75–17.71]	3.26	3.15–3.34	32.80	32.17–33.40	Subgroup distributional interval
36–50 years	15,598	13.20 [8.58–19.40]	3.57	3.46–3.70	35.10	34.50–36.10	Subgroup distributional interval
51–65 years	11,144	14.16 [9.20–20.90]	3.78	3.62–3.97	39.63	38.50–40.80	Subgroup distributional interval
Winter	13,076	11.64 [7.77–17.40]	3.25	3.06–3.34	35.55	34.64–36.31	Subgroup distributional interval
Spring	11,674	11.40 [7.38–17.60]	3.30	3.14–3.40	34.92	34.30–36.16	Subgroup distributional interval
Summer	12,192	14.50 [9.53–20.50]	4.11	3.92–4.25	35.60	34.70–36.50	Subgroup distributional interval
Autumn	11,884	13.70 [8.84–19.80]	3.41	3.24–3.52	35.60	34.90–36.72	Subgroup distributional interval

These limits represent nonparametric 2.5th–97.5th percentile distributional limits from a clinically pre-filtered routine laboratory dataset. They are not healthy-volunteer reference limits, biological normal ranges, clinical sufficiency thresholds, treatment thresholds, or clinical decision limits. The Tukey 1.5 × IQR exclusion approach was used only for the overall sensitivity analysis and did not define the primary analytical dataset. 25(OH)D, 25-hydroxyvitamin D; CI, confidence interval; IQR, interquartile range.

**Table 4:** Adjusted associations of sex, age group, and season with serum 25(OH)D and vitamin D deficiency.

Predictor	Reference Category	GMR for Serum 25(OH)D (95% CI)	*p*-Value	Adjusted OR for Vitamin D Deficiency (95% CI)	*p*-Value
Female	Male	0.769 (0.760–0.778)	<0.001	1.56 (1.49–1.63)	<0.001
18–35 years	51–65 years	0.855 (0.844–0.866)	<0.001	1.72 (1.63–1.82)	<0.001
36–50 years	51–65 years	0.940 (0.927–0.953)	<0.001	1.25 (1.18–1.32)	<0.001
Winter	Summer	0.833 (0.821–0.845)	<0.001	1.65 (1.56–1.76)	<0.001
Spring	Summer	0.820 (0.808–0.832)	<0.001	1.68 (1.58–1.79)	<0.001
Autumn	Summer	0.933 (0.920–0.947)	<0.001	1.16 (1.09–1.23)	<0.001

Serum 25(OH)D was modeled after natural-log transformation, and results are reported as geometric mean ratios. Vitamin D deficiency was defined as serum 25(OH)D < 20 ng/mL and modeled using binary logistic regression. Reference categories were male sex, age 51–65 years, and summer. Adjusted models included sex, age group, and season. These models describe adjusted associations and should not be interpreted as causal effects. 25(OH)D, 25-hydroxyvitamin D; CI, confidence interval; GMR, geometric mean ratio; OR, odds ratio.

## Data Availability

The data supporting the findings of this study are available from the corresponding author upon reasonable request due to ethical restrictions.
